# Placebo use in vaccine trials: Recommendations of a WHO expert panel

**DOI:** 10.1016/j.vaccine.2014.04.022

**Published:** 2014-08-20

**Authors:** Annette Rid, Abha Saxena, Abdhullah H. Baqui, Anant Bhan, Julie Bines, Marie-Charlotte Bouesseau, Arthur Caplan, James Colgrove, Ames Dhai, Rita Gomez-Diaz, Shane K. Green, Gagandeep Kang, Rosanna Lagos, Patricia Loh, Alex John London, Kim Mulholland, Pieter Neels, Punee Pitisuttithum, Samba Cor Sarr, Michael Selgelid, Mark Sheehan, Peter G. Smith

**Affiliations:** a Department of Social Science, Health & Medicine, King's College London, Strand, London WC2R 2LS, United Kingdom; b Knowledge, Ethics and Research, World Health Organization, Geneva, Switzerland; c International Center for Maternal and Newborn Health, Johns Hopkins Bloomberg School of Public Health, Baltimore, MD, United States; d Ethical, Social and Cultural Program for Global Health, University of Toronto, Toronto, ON, Canada; e Murdoch Childrens Research Institute, University of Melbourne, Parkville, VIC, Australia; f Service Delivery and Safety, World Health Organization, Geneva, Switzerland; g Division of Medical Ethics, NYU School of Medicine, New York, NY, United States; h Mailman School of Public Health, Columbia University, New York, NY, United States; i Steve Biko Centre for Bioethics, University of Witwatersrand, Johannesburg, South Africa; j Instituto Mexicano del Seguro Social, Centro Médico Nacional Siglo XXI, Ciudad de Mexico, D.F., Mexico; k Ethical, Social and Cultural Program for Global Health, St. Michael's Hospital and University of Toronto, Toronto, ON, Canada; l Christian Medical College, Vellore, Tamil Nadu, India; m Hospital de Niños Roberto del Río, Santiago de Chile, Chile; n Melbourne Law School, University of Melbourne, Carlton, VIC, Australia; o Department of Philosophy, Carnegie Mellon University, Pittsburgh, PA, United States; p London School of Hygiene and Tropical Medicine, London, United Kingdom; q Vaccine Advice BVBA, Zoersel, Belgium; r Faculty of Tropical Medicine, Mahidol University, Bangkok, Thailand; s Ministry of Health and Social Action 1, Dakar-Fann, Senegal; t Centre for Human Bioethics, Monash University, Monash, VIC, Australia; u The Ethox Centre, University of Oxford, Oxford, United Kingdom; v Medical Research Council (MRC) Tropical Epidemiology Group, London School of Hygiene and Tropical Medicine, London, United Kingdom

**Keywords:** Vaccine trials, Trial design, Ethics, Placebo controls, Risk, International research

## Abstract

Vaccines are among the most cost-effective interventions against infectious diseases. Many candidate vaccines targeting neglected diseases in low- and middle-income countries are now progressing to large-scale clinical testing. However, controversy surrounds the appropriate design of vaccine trials and, in particular, the use of unvaccinated controls (with or without placebo) when an efficacious vaccine already exists. This paper specifies four situations in which placebo use may be acceptable, provided that the study question cannot be answered in an active-controlled trial design; the risks of delaying or foregoing an efficacious vaccine are mitigated; the risks of using a placebo control are justified by the social and public health value of the research; and the research is responsive to local health needs. The four situations are: (1) developing a locally affordable vaccine, (2) evaluating the local safety and efficacy of an existing vaccine, (3) testing a new vaccine when an existing vaccine is considered inappropriate for local use (e.g. based on epidemiologic or demographic factors), and (4) determining the local burden of disease.

## Highlights

• Placebo controls may be acceptable even when an efficacious vaccine exists, in the following four possible situations:
• When developing a locally affordable vaccine.
• When evaluating the local safety and efficacy of an existing vaccine.
• When testing a new vaccine when an existing vaccine is not considered appropriate locally.
• When determining the local burden of disease.

## 1 Background

Infectious diseases continue to pose a tremendous burden of disease worldwide, especially in low- and middle-income countries (LMICs) [1]. Vaccines exist for many common infectious diseases, and they are among the most cost-effective means of reducing this burden. However, for many debilitating and life-threatening infectious diseases in LMICs, vaccines either do not exist, or they are insufficiently efficacious^1^ or unavailable to most of the population due to high cost.

Many vaccines targeting diseases prevalent in LMICs are currently under development. As investigators and sponsors plan large-scale clinical trials to test the safety and efficacy of these new vaccines, important ethical issues can arise in trial design, particularly around the use of a placebo control arm when an efficacious vaccine already exists.

Randomised, placebo-controlled trials are widely considered the gold standard for evaluating the safety and efficacy of a new vaccine. In these trials, participants are randomized to receive either the vaccine under investigation or a placebo (i.e. an inert substance, such as a saline injection). Randomisation and the use of placebo interventions are designed to control for confounding effects, such that significant differences in disease incidence or adverse effects between the vaccine and control groups can likely be attributed to the vaccine. However, randomised, placebo-controlled trial designs often raise ethical concerns when participants in the control arm are deprived of an existing vaccine. Furthermore, testing a new vaccine against placebo is scientifically and ethically fraught when the hypothesis being tested is whether an experimental vaccine is more efficacious than one already in use in the same or in other settings.

Currently, there is insufficient and inconsistent guidance on how to evaluate the use of placebo controls in vaccine trials. Most ethical guidelines for research do not address vaccine trials specifically; and, in those that do, the guidance regarding placebo use is limited [2], [3]. Moreover, general ethical guidelines for research – authored by both national and international bodies – offer conflicting guidance on the use of placebo controls [4], [5], [6], [7], [8], [9], [10], [11]. Some guidelines call for exclusion of placebo use altogether when there is a proven or established effective intervention against the condition under study [10]. Others allow placebo use, provided the risks of withholding or delaying the existing intervention are either negligible or there are compelling methodological reasons for including a placebo arm in the trial [4], [5], [7], [8], [9].

Yet, the level of risk deemed acceptable when there are compelling reasons for placebo use varies greatly. Most guidelines allow no more than minimal risks, excluding risks of serious or irreversible harm [4], [5], [9] or allowing placebo use only in the case of self-limiting disease [7]. In contrast, others set no explicit risk limit in research that is relevant to the local population [8]. Some documents openly acknowledge the “unresolved, or unresolvable, conflict” around placebo use [5], a situation that has led influential regulators to disregard ethical guidance that they deem overly restrictive [12].

The lack of consistent guidance on the use of placebo controls raises significant ethical concern. On the one hand, investigators and sponsors may avoid conducting placebo-controlled trials when an efficacious vaccine exists, even if such trials are scientifically necessary and potentially justifiable. On the other hand, a lack of clear guidance may result in the conduct of placebo-controlled trials that are ultimately unethical.

## 2 WHO expert consultation

Against this backdrop, the WHO Department of Ethics and Social Determinants convened an expert consultation to provide recommendations on the use of placebo controls in vaccine trials in cases where an efficacious vaccine already exists. The focus was on large-scale clinical trials that test vaccines in Phases III and IV of development (i.e. where preliminary testing of safety and immunogenicity, and sometimes efficacy, has been completed in Phase I and II trials). The panel, consisting of 20 experts from 11 countries, met to discuss relevant issues and develop recommendations in consultation with key stakeholders in international vaccine research (Appendix). The present paper develops the discussion and conclusions from that meeting [13].

## 3 General ethical considerations

Given the high burden of infectious diseases, especially in LMICs, there is an ethical imperative to develop and test new vaccines. The recommendations from the panel therefore aim to facilitate the conduct of vaccine research that is ethical, scientifically valid, and designed to meet important public health needs.

While this paper focuses specifically on the use of placebo controls, similar considerations apply to open designs in which a placebo is not used, but an unvaccinated control group is included. The following recommendations assume that other common requirements for ethical research are respected [4], [5]. In particular: Investigators and sponsors consult and collaborate with local stakeholders in all phases of the research; research participants, or their legal representatives, give voluntary and informed consent to study participation; participants are free to withdraw from research at any time, for any reason, without penalty; the research addresses an important health problem and is responsive to local health needs; the study design used minimizes risks and enhances potential clinical benefits for participants; the benefits and burdens of the research are justly distributed; and sponsors, in consultation with national or local authorities, make provisions to ensure reasonable post-trial access to interventions proven most efficacious to the population from which the research participants were drawn.

## 4 Ethical framework for placebo use in vaccine trials

To navigate the difficult ethical terrain of using placebo controls in vaccine trials, it is helpful to identify the conditions under which placebo use is clearly acceptable and clearly unacceptable. The following considerations assume that placebo interventions (e.g. subcutaneous injections of saline solution) themselves pose negligible risks.

Placebo use in vaccine trials is clearly *acceptable* when (a) no efficacious and safe vaccine exists and (b) the vaccine under consideration is intended to benefit the population in which the vaccine is to be tested. In this situation, a placebo-controlled trial addresses the locally relevant question regarding the extent to which the new vaccine is better than nothing, and participants in the placebo arm of the trial are not deprived of the clinical benefits of an existing efficacious vaccine.

Placebo use in vaccine trials is clearly *unacceptable* when (a) a highly efficacious and safe vaccine exists and is currently accessible in the public health system of the country in which the trial is planned and (b) the risks to participants of delaying or foregoing the available vaccine cannot be adequately minimized or mitigated (e.g. by providing counselling and education on behavioural disease prevention strategies, or ensuring adequate treatment for the condition under study to prevent serious harm). In this situation, a placebo-controlled trial would not address a question that is relevant in the local context, namely how the new vaccine compares to the one that is currently in use, and participants would be exposed to unacceptable levels of risk from delaying or foregoing a safe and effective vaccine that is accessible through the public health system.

Between these two poles, the use of placebo controls in vaccine trials may be justified even when an efficacious vaccine exists, provided the risk-benefit profile of the trial is acceptable. This applies to situations where the existing vaccine is available through the local public health system, as well as to situations where the existing vaccine is not available locally, or it is only available on the private market. Specifically, the risk-benefit profile of a placebo-controlled vaccine trial may be acceptable when (1) the study question cannot be answered with an active-controlled trial design; and (2) the risks of delaying or foregoing an existing efficacious vaccine are adequately minimized or mitigated; and (3) the use of a placebo control is justified by the potential public health or social value of the research; and (4) the research is responsive to local health needs. Importantly, and contrary to many of the existing ethical guidelines on placebo use [4], [5], [7], [9], the acceptable risks of withholding or delaying administration of an existing vaccine in the placebo arm of vaccine trials may be greater than minimal when the above conditions are met.

## 5 Possible situations of acceptable placebo use

The following four scenarios specify situations between the two poles of clearly acceptable and clearly unacceptable placebo use in vaccine trials. In these situations, the use of a placebo control may be acceptable when an efficacious vaccine exists, provided the above four conditions are met.

### 5.1 Developing a locally affordable vaccine

A new, low-cost vaccine is tested against a placebo because the existing vaccine – although known to be, or likely to be, efficacious in the trial country – is inaccessible in that country's public health system. Accessibility may be hindered by limitations in a health system's ability to provide adequate support in areas including administration, financing, production, distribution and infrastructure. Furthermore, there should be strong reasons to believe that the existing vaccine is likely to remain inaccessible in the future, and the new vaccine, if proven efficacious, will not be subject to the same limitations that have prevented use of the existing vaccine. In this situation, a placebo arm might be justified to assess how effective the trial vaccine is compared to no vaccine.

Example Diarrhoeal disease due to rotavirus infections is a major cause of morbidity and mortality in India. Two efficacious rotavirus vaccines to protect against severe rotavirus gastroenteritis exist [14], but their cost remains prohibitive in many LMICs and experts debate the likely local efficacy of the vaccines in some countries. Although the existing vaccines were licensed in India, they were not – nor were they planned to be – introduced into the national immunization programme for reasons of cost and a lack of data on vaccine efficacy in Indian children. An Indian vaccine company and a consortium of partner organizations conducted a placebo-controlled trial of a new low-cost vaccine that was based on a strain of rotavirus isolated in India and targeted at infants in India and other LMICs [15]. To mitigate risk in the placebo arm, the trial design included close monitoring of all participants to identify and treat cases of gastroenteritis as early as possible. This system of active surveillance and early evaluation and treatment significantly reduced the mortality risk of severe rotavirus gastroenteritis in the study population.

### 5.2 Evaluating the local safety and efficacy of an existing vaccine

An existing vaccine is tested against a placebo to evaluate its safety and efficacy in the trial country prior to uptake and introduction into the health system. As there is sometimes insufficient information about the safety and efficacy of existing vaccines in different settings, the status of an existing vaccine as “established effective” in a particular local context may need to be determined.

Example A conjugate vaccine against pneumococcal disease, based on seven serotypes, had been developed and was included in the routine vaccination programme of many high-income countries. Although the vaccine was expected to protect against pneumococcal disease in Africa, it was unclear if the seven included serotypes were appropriate for use on this continent. In addition, there was uncertainty about the burden of disease in Africa, particularly pneumococcal pneumonia, where a causative agent cannot be isolated in most cases of pneumonia. Two additional serotypes were added to the existing seven-valent vaccine and the new vaccine was tested in a placebo-controlled trial in The Gambia, evaluating the impact of the vaccine against radiologically-proven pneumonia [16]. The trial showed 37% protection against radiological pneumonia, a finding that has been important in promoting the use of pneumococcal vaccines in many LMICs.

### 5.3 Testing a new vaccine when an existing vaccine is considered inappropriate for local use

A new vaccine is tested against a placebo because scientific experts or health officials in the trial country have determined that the existing vaccine should not be used in the national vaccination programme because it is not considered to be sufficiently efficacious due to local epidemiologic, demographic, environmental, or logistical factors. For example, the existing vaccine may provide inadequate levels of protection, the protection may not be durable, or it may require multiple vaccinations whose timely administration cannot be ensured under local circumstances. In this situation, a placebo arm is scientifically necessary in order to obtain sufficient information on the new vaccine's efficacy or effectiveness. An existing vaccine may also be considered inappropriate for local use when it is unacceptable to a population, including the potential study participants in the trial country, based on deeply held cultural or religious values (e.g. some religions do not approve of the use of bovine or porcine derived products except in emergency situations [17], and several vaccines contain such products).

Example Three new candidate vaccines against leprosy were tested in a trial in India. Previous evidence indicated that the existing BCG vaccination offered about 20–30% protection against leprosy locally. However, Indian health officials did not consider this level of protection sufficiently high to justify deploying the vaccine through the national immunization programme. The five-arm leprosy vaccine trial therefore included two control arms, with one arm receiving the BCG vaccine and one receiving a placebo. The trial confirmed the low efficacy of the BCG vaccine and demonstrated a ∼65% protection for two of the three new vaccines [18]. For reasons that are unclear, neither of the two efficacious vaccines was subsequently included in Indian public health programmes.

### 5.4 Determining the local burden of disease

An existing vaccine is tested against a placebo because the public health significance of the vaccine's introduction in the trial country – that is, the vaccine's effect on the burden of morbidity and mortality due to the condition(s) against which the vaccine protects – is unknown or uncertain. Comparison with a placebo yields information on the expected public health impact of introducing the existing vaccine, thereby facilitating informed decisions by public health officials.

Example Most studies had found low rates of *Haemophilus influenzae* type b (Hib) disease in Asia, and few Asian countries therefore included Hib vaccine into their routine immunization programmes. Yet it was unclear whether Hib disease truly is rare, or whether many cases simply remain undetected. A so-called “vaccine probe study” was conducted in Indonesia to estimate the local burden of preventable Hib pneumonia and meningitis by randomizing children in ∼800 hamlets to receive either normal vaccinations or normal vaccinations and the existing Hib vaccine [19]. Hib vaccine did not prevent the great majority of pneumonia cases and the results did not support a major role for Hib vaccine in overall pneumonia-prevention programmes. However, the study identified high incidences of Hib meningitis and pneumonia which was used to support the inclusion of Hib vaccine in routine infant immunization programmes in many Asian countries.

## 6 Weighing alternative trial designs

When evaluating the acceptability of using a placebo control in vaccine trials, it is essential for investigators, sponsors, research ethics committees (RECs), and relevant other parties to consider alternative trial or study designs that might minimize risks and enhance potential clinical benefits for participants. For example, in situations where a vaccine is known to be efficacious but the local burden of disease is uncertain, investigators and others should first evaluate study designs other than a placebo-controlled trial that might allow determining the burden of disease (e.g. measuring the burden of gastroenteritis before and after introducing rotavirus vaccines in Latin America Desai, Oliveira [20]). Furthermore, when a placebo-controlled trial is thought to be necessary, it is important to consider a design that combines the investigational vaccine or placebo with a routine vaccination and thus avoids giving participants an additional injection (e.g. pneumococcus vaccine trial in the Gambia where the experimental vaccine or placebo was mixed with the DTP–Hib vaccine [16]).

Investigators and others should also consider enhancing the potential scientific and social value of vaccine trials by including additional study arms. For example, when the benefits of an existing vaccine are uncertain in the local population, testing a new vaccine against both a placebo and the existing vaccine would adequately answer the study question, while also providing evidence to evaluate the existing vaccine under local circumstances (e.g. leprosy vaccine trial in India [18]). However, trials that include an existing vaccine as a comparator typically require larger sample sizes and hence are more resource intensive than trials using a placebo control alone. The expense, time and trial infrastructure requirements entailed by active comparator trials may discourage investigators or sponsors from conducting them, thereby delaying the delivery of new vaccines in populations that may need them most urgently.

Finally, as part of the discussions around trial design, investigators, sponsors and RECs should consider different types of “placebo” interventions. Rather than using a true placebo control (i.e. an inert substance), it may be appropriate to use a vaccine against a disease that is not the focus of the trial (e.g. an ongoing malaria vaccine trial provides non-malaria vaccines to participants in the control arm [21], [22]). The motivation for using these types of “placebos” is to benefit participants in the control arm and avoid giving an injection with an inert substance. However, this motivation undescores the importance of ensuring that the comparator vaccine(s) are proven to be beneficial in the study population. Furthermore, it is important to recognize that trials using such “placebos” may provide a less perfect control if the effects of the comparator vaccine(s) confound the evaluation of the risk-benefit profile of the experimental vaccine. For this reason, use of such “placebos” may also be less acceptable to regulators or public health authorities and potentially delay approval or adoption of a new vaccine.

## 7 Procedural requirements

Applying the above ethical framework requires that investigators, sponsors, local communities, RECs, drug/vaccine regulators, public health authorities, policy-makers, and other relevant parties make complex normative and empirical judgments. All of these stakeholders therefore have an obligation to ensure that decisions about vaccine trial design, and especially the use of placebo controls when an efficacious vaccine exists, are made based on the best available evidence and under consideration of all relevant reasons.

### 7.1 Research ethics review

All vaccine trials should undergo REC review prior to enrolling participants. Investigators and sponsors are responsible for submitting a research protocol that gives a clear ethical justification for the proposed trial design in line with the above considerations and presents relevant empirical evidence in a balanced and comprehensible way. The protocol should explain clearly both the scientific justification for and the social value of using a placebo-controlled design and discuss the relative merits of alternative trial designs. The justification for not using an existing vaccine as a comparator should include discussion of the acceptability, availability, and accessibility of the existing vaccine for the prospective trial population. It must be clear that the study question cannot be answered in an active-controlled trial in the target population.

Furthermore, the protocol should provide evidence to support all empirical claims. This includes relevant evidence from previous clinical and non-clinical studies; evidence from consultation with experts (e.g. to support claims about the local safety and efficacy of an existing vaccine); evidence from consultation with local stakeholders (e.g. to show that the study infrastructure is appropriate); and evidence from formative surveys or interviews (e.g. to demonstrate local acceptability of the vaccine if found effective). Furthermore, when an existing vaccine is not used in the trial country because of financial, logistical, social, religious or cultural barriers to access, the protocol should provide evidence that these barriers are unlikely to be overcome in the foreseeable future, and that the same barriers would not obstruct use of the experimental vaccine should it prove efficacious.

RECs are responsible for evaluating research protocols and carefully scrutinizing ethical arguments, as well as the evidence to support empirical claims. RECs should therefore either have members who are knowledgeable about vaccine research and vaccine policy, or they should be open to consulting with independent experts in this area. Where necessary, sponsors should support expansion of RECs’ capacity. For instance, independent experts may present available data to RECs to guide them when evaluating the adequacy of any local evidence. Importantly, experts can be available for advice and discussion without participating in the REC's actual decision-making process. In some cases, an internationally coordinated “pre-review” of the study protocol could support local RECs by mapping the relevant ethical issues posed by the study. This could be particularly helpful when trials are conducted in countries where the local ethics review system remains remains underdeveloped. Finally, to help protect and promote trust and confidence in research oversight, RECs should record their justification for approving a placebo-controlled trial when an efficacious vaccine exists, and ideally make it publicly accessible. Study sponsors could also make this justification publicly available in clinical trial registries.

### 7.2 Consultation and collaboration with local stakeholders

Early and ongoing consultation and collaboration between sponsors and host country stakeholders in government and civil society are essential. Before planning a trial, sponsors should consult with relevant local stakeholders both about the barriers to use of any existing vaccine(s) and the necessary and sufficient conditions for uptake of a new vaccine. Sponsors should pay particular attention to political, social and practical issues that may affect uptake. This may include formative surveys or interviews (e.g. to assess the political and economic aspects of the local health system). Sponsors and investigators are responsible for communicating appropriately about trial risks with all stakeholders. Risk assessments should be based on the available evidence and local context, and they should include the risks of delaying or not conducting the trial. During the planning and review of vaccine trials, sponsors and investigators should be accessible to local stakeholders to discuss the often complicated scientific and epidemiological questions that are relevant to ethical decision-making. There is no single model for how such consultation should take place, it may be ad hoc and trial-specific. Where necessary, appropriate structures for ethical discussions should be created. Finally, health authorities should facilitate ethical discussions among all involved parties prior to approving a vaccine trial under their jurisdiction, and should make the outcome of these discussions available to everyone interested.

## 8 Conclusion

Vaccine trial design can raise challenging ethical questions, especially regarding the use of placebo controls when an efficacious vaccine exists. This paper presents an ethical framework for addressing questions concerning placebo-controlled trials, as developed by a recent WHO expert panel. The framework sets out the conditions under which placebo use is clearly acceptable and clearly unacceptable in vaccine trials. It then specifies four situations in which the use of placebo controls may be ethically justified even when an efficacious vaccine exists. In these situations, it is necessary that the study question cannot be answered in an active-controlled trial design; that the risks of delaying or foregoing the efficacious vaccine are adequately mitigated; that the risks of using a placebo control are justified by the social or public health value of the research; and that the research is responsive to local health needs. The ultimate judgement about the acceptability of using a placebo control when an efficacious vaccine exists will depend on the specifics of the given trial. It is therefore critical that investigators and sponsors develop the design of vaccine trials in close collaboration with host country stakeholders, and that RECs and others thoroughly evaluate study protocols based on the available evidence and all relevant reasons. It is our hope that these recommendations will help to ensure that participants in vaccine trials are protected from unjustifiable risks, while facilitating the conduct of valuable and urgently needed vaccine research.

## Author contributions

Annette Rid, Abha Saxena and Peter Smith drafted the initial manuscript based on the WHO meeting report. All authors reviewed and revised the manuscript, and approved the final manuscript as submitted.

## Funding

The WHO Expert Consultation was supported by 10.13039/100005624PATH, a non-profit organization funded by the Bill & Melinda Gates Foundation. Annette Rid received funding from the People Programme (Marie Curie Actions) of the European Union's Seventh Framework Programme (FP7/2007-2013) under 10.13039/501100000783REA grant agreement no. 301816. Peter G. Smith receives support from the MRC and DiFD (MR/K012126/1). Mark Sheehan is grateful for the support of the Oxford NIHR Biomedical Research Centre.

## Conflicts of interest statement

Several authors of this paper have been involved in placebo-controlled vaccine trials that were conducted in situations in which a vaccine already existed that was at least partially efficacious against the conditions under study.

## Footnotes

1 We use the term “efficacious” to indicate a vaccine that reduces the incidence of the target disease. This includes vaccines that may have high or low efficacy.
