# Supplementary material for: Placebo use in vaccine trials: Recommendations of a WHO expert panel
Source: Vaccine. 2014 Aug 20;32(37):4708–12. doi: 10.1016/j.vaccine.2014.04.022 (PMC4157320; doi:10.1016/j.vaccine.2014.04.022)
Supplement: Supplementary file 1 [file mmc1.docx]

APPENDIX

**List of participants at the WHO Expert Consultation on Placebo Use in Vaccine Trials, Annecy, 17-18 January, 2013.**

| **Experts** | **Affiliations** |
| --- | --- |
| Baqui, Abdullah H. | Director, International Center for Maternal and Newborn Health  Johns Hopkins Bloomberg School of Public Health  Baltimore, Maryland, USA  E-mail: [abaqui@jhsph.edu](mailto:abaqui@jhsph.edu) |
| Bhan, Anant | Global Health and Bioethics Consultant  Pune, Maharashtra, India;  Bioethicist,  Ethical, Social and Cultural Program for Global Health  Sandra Rotman Centre, Toronto, Canada  E-mail: [anantbhan@gmail.com](mailto:anantbhan@gmail.com) |
| Bines, Julie | The University of Melbourne  Royal Children’s Hospital  Parkville, Victoria, Australia  E-mail: [julie.bines@rch.org.au](mailto:julie.bines@rch.org.au) |
| Caplan, Arthur | NYU School of Medicine  Division of Medical Ethics  New York, New York, USA  E-mail: [Arthur.Caplan@nyumc.org](mailto:Arthur.Caplan@nyumc.org) |
| Colgrove, James | Mailman School of Public Health  Department of Sociomedical Sciences  Columbia University  New York, New York, USA  E-mail: [jc988@columbia.edu](mailto:jc988@columbia.edu) |
| Dhai, Ames | Steve Biko Centre for Bioethics  University of the Witwatersrand  Johannesburg, South Africa  E-mail: [Amaboo.Dhai@wits.ac.za](mailto:Amaboo.Dhai@wits.ac.za) |
| Gomez Diaz, Rita | Instituto Mexicano del Seguro Social  Unidad de Investigación en Epidemiología Clínica  Hospital de Especialidades  Centro Médico Nacional Siglo XXI  Mexico City, Mexico  E-mail: [ritagomezdiaz@yahoo.com.mx](mailto:ritagomezdiaz@yahoo.com.mx) |
| Green, Shane | Ethical, Social and Cultural Program for Global Health  Sandra Rotman Centre  University Health Network and University of Toronto  Toronto, Canada  E-mail: [shane.green@srcglobal.org](mailto:shane.green@srcglobal.org) |
| Kang, Gagandeep | Christian Medical College  Vellore, Tamil Nadu, India  E-mail: [gkang@cmcvellore.ac.in](mailto:gkang@cmcvellore.ac.in) |
| Lagos, Rosanna | Centro para Vacunas en Desarrollo-Chile (CVD-Chile)  Hospital de Niños Roberto del Río  Santiago, Chile  E-mail: [rosanna.lagos@adsl.tie.cl](mailto:rosanna.lagos@adsl.tie.cl) |
| London, Alex John  (absent) | Carnegie Mellon University Pittsburgh, Pennsylvania, USA  E-mail: [alexJohnLondon@gmail.com](mailto:AlexJohnLondon@gmail.com) |
| Mulholland, Kim | London School of Hygiene and Tropical Medicine  London, United Kingdom  E-mail: [kim.mulholland@lshtm.ac.uk](mailto:kim.mulholland@lshtm.ac.uk) |
| Neels, Pieter | Federal Agency for Medicinal and Health Products (FAMHP)  Brussels, Belgium  E-mail: [pieter.neels@fagg-afmps.be](mailto:pieter.neels@fagg-afmps.be) |
| Pitisuttithum, Punnee | Faculty of Tropical Medicine  Mahidol University  Bangkok, Thailand  E-mail: [tmppt@mahidol.ac.th](mailto:tmppt@mahidol.ac.th) |
| Pratt, Douglas | Center for Biologics Evaluation and Research (CBER)  Food and Drug Administration (FDA)  Rockville, Maryland, USA  E-mail: [Douglas.Pratt@fda.hhs.gov](mailto:Douglas.Pratt@fda.hhs.gov) |
| Rid, Annette | Department of Social Science, Health & Medicine  King's College London  London, United Kingdom  E-mail: annette.rid@kcl.ac.uk |
| Sarr, Samba Cor | Ministry of Health and Social Action 1  Dakar-Fann, Senegal  E-mail: [bathie65@yahoo.fr](mailto:bathie65@yahoo.fr) |
| Selgelid, Michael | Centre for Human Bioethics  Monash University  Monash, Victoria, Australia  E-mail: [michael.selgelid@monash.edu](mailto:michael.selgelid@monash.edu) |
| Sheehan, Mark | Oxford Biomedical Research Centre Ethics Fellow  The Ethox Centre  University of Oxford  Oxford, United Kingdom  E-mail: [mark.sheehan@ethox.ox.ac.uk](mailto:mark.sheehan@ethox.ox.ac.uk) |
| Smith, Peter G | Medical Research Council Tropical Epidemiology Group  London School of Hygiene and Tropical Medicine  London, United Kingdom  E-mail: [Peter.Smith@lshtm.ac.uk](mailto:Peter.Smith@lshtm.ac.uk) |

| **Observers*** | **Affiliations** |
| --- | --- |
| Boslego, John | Director, Vaccine Development  PATH  Seattle, Washington, USA  E-mail: [jboslego@path.org](mailto:jboslego@path.org) |
| Danagher, Pamela | Bill & Melinda Gates Foundation  Seattle, Washington, USA E-mail: [falconpointconsulting@gmail.com](mailto:falconpointconsulting@gmail.com) |
| De Menezes Martins, Reinaldo | Consultor Científico Senior  Bio-Manguinhos/Fiocruz  Rio de Janeiro, Brazil  Email: [Rmenezes@bio.fiocruz.br](mailto:Rmenezes@bio.fiocruz.br) |
| Dhere, Rajeev | Serum Institute of India Ltd  Pune, Maharashtra, India  E-mail: [rajeev.dhere@seruminstitute.com](mailto:rajeev.dhere@seruminstitute.com) |
| Fix, Alan | PATH  Seattle, Washington, USA  E-mail: [fixa@path.org](mailto:fixa@path.org) |
| Fix, Jonathan | Independent  E-mail: [jonathanfix@verizon.net](mailto:jonathanfix@verizon.net) |
| Kulkarni, Prasad | Serum Institute of India Ltd  Pune, Maharashtra, India  E-mail: [drpsk@seruminstitute.com](mailto:drpsk@seruminstitute.com) |
| Muhyini, Etna | Staff of Clinical Trial Department  Bio Farma  Bandung, Indonesia  E-mail: [etna@biofarma.co.id](mailto:etna@biofarma.co.id) |
| Mulia Sari, Rini | Staff of Clinical Trial Department  Bio Farma  Bandung, Indonesia  E-mail: [rini.mulia@biofarma](mailto:rini.mulia@biofarma).co.id |
| Pagliusi, Sonia | Executive Secretary  Developing Countries Vaccine Manufacturers Network (DCVMN) International  Nyon, Switzerland  E-mail: [s.pagliusi@dcvmn.org](mailto:s.pagliusi@dcvmn.org) |

* Day 1 of the Consultation only (background presentations and discussion of case studies)

| **WHO Secretariat** | **Affiliations** |
| --- | --- |
| Bouësseau, Marie-Charlotte | Department of Ethics and Social Determinants of Health  World Health Organization  Geneva, Switzerland  E-mail: [bouesseaum@who.int](mailto:bouesseaum@who.int) |
| Krech, Rüdiger | Department of Ethics and Social Determinants of Health  World Health Organization  Geneva, Switzerland  E-mail: [krechr@who.int](mailto:krechr@who.int) |
| Moorthy, Vasee | WHO-UNAIDS HIV Vaccine Initiative  World Health Organization  Geneva, Switzerland  E-mail: [moorthyv@who.int](mailto:moorthyv@who.int) |
| Nishioka, Sergio | Department of Essential Medicines and Pharmaceutical Policies  World Health Organization  Geneva, Switzerland  E-mail: [nishiokas@who.int](mailto:nishiokas@who.int) |
| Reis, Andreas | Department of Ethics and Social Determinants of Health  World Health Organization  Geneva, Switzerland  E-mail: [reisa@who.int](mailto:reisa@who.int) |
| Saxena, Abha | Department of Ethics and Social Determinants of Health  World Health Organization  Geneva, Switzerland  E-mail: [saxenaa@who.int](mailto:saxenaa@who.int) |
| **WHO Interns** | |
| Loh, Patricia | Department of Ethics and Social Determinants of Health  World Health Organization  Geneva, Switzerland |
| Song, Hyun | Department of Ethics and Social Determinants of Health  World Health Organization  Geneva, Switzerland |
